# Plasma Steroid Profiles in Individuals With Class II/III Obesity: Association With Weight Loss After Metabolic Surgery

**DOI:** 10.1210/jendso/bvaf151

**Published:** 2025-09-22

**Authors:** Mari Ibusuki, Kohta Nakatani, Yayoi Matsuda, Hironobu Umakoshi, Maki Yokomoto-Umakoshi, Hiroki Takayanagi, Ryuichi Sakamoto, Tetsuro Kawazoe, Eiji Oki, Tomoharu Yoshizumi, Yoshihiro Izumi, Takeshi Bamba, Yoshihiro Ogawa

**Affiliations:** Department of Medicine and Bioregulatory Science, Graduate School of Medical Sciences, Kyushu University, Fukuoka 812-8582, Japan; Division of Metabolomics, Medical Research Center for High Depth Omics, Medical Institute of Bioregulation, Kyushu University, Fukuoka 812-8582, Japan; Department of Medicine and Bioregulatory Science, Graduate School of Medical Sciences, Kyushu University, Fukuoka 812-8582, Japan; Department of Medicine and Bioregulatory Science, Graduate School of Medical Sciences, Kyushu University, Fukuoka 812-8582, Japan; Department of Medicine and Bioregulatory Science, Graduate School of Medical Sciences, Kyushu University, Fukuoka 812-8582, Japan; Department of Medicine and Bioregulatory Science, Graduate School of Medical Sciences, Kyushu University, Fukuoka 812-8582, Japan; Department of Medicine and Bioregulatory Science, Graduate School of Medical Sciences, Kyushu University, Fukuoka 812-8582, Japan; Department of Surgery and Science, Graduate School of Medical Sciences, Kyushu University, Fukuoka 812-8582, Japan; Department of Surgery and Science, Graduate School of Medical Sciences, Kyushu University, Fukuoka 812-8582, Japan; Department of Surgery and Science, Graduate School of Medical Sciences, Kyushu University, Fukuoka 812-8582, Japan; Division of Metabolomics, Medical Research Center for High Depth Omics, Medical Institute of Bioregulation, Kyushu University, Fukuoka 812-8582, Japan; Division of Metabolomics, Medical Research Center for High Depth Omics, Medical Institute of Bioregulation, Kyushu University, Fukuoka 812-8582, Japan; Department of Medicine and Bioregulatory Science, Graduate School of Medical Sciences, Kyushu University, Fukuoka 812-8582, Japan

**Keywords:** class II/III obesity, severe obesity, steroid metabolites, metabolic surgery, laparoscopic sleeve gastrectomy, 17α-hydroxypregnenolone

## Abstract

**Context:**

Although steroid metabolism is altered in individuals with obesity, comprehensive profiles of steroid metabolites remain unexplored, some of which may be related to weight loss after metabolic surgery.

**Objective:**

We aimed to characterize comprehensive steroid profiles in individuals with class II/III obesity (body mass index ≥35 kg/m^2^) and identify metabolite(s) related to weight loss outcomes after laparoscopic sleeve gastrectomy (LSG).

**Methods:**

Using liquid chromatography–tandem mass spectrometry, we measured 27 plasma steroid metabolites in individuals with class II/III obesity (n = 93), healthy controls (n = 15), and those after LSG (n = 20).

**Results:**

Discriminant analysis revealed distinct steroid profiles between individuals with class II/III obesity and healthy controls, with statistical significance for 9 metabolites in men (n = 53) and 11 in premenopausal women (n = 44). One year after LSG, the insufficient and sufficient weight loss groups (percent total weight loss (%TWL) < 20%; n = 10 and %TWL ≥ 20%; n = 26) showed distinct preoperative steroid profiles. Preoperative 17α-hydroxypregnenolone (17α-OHPreg) levels, which were lower in individuals with class II/III obesity, were the most significant factor contributing to this distinction, and remained significantly lower in the insufficient weight loss group even after adjusting for confounders (*P* = .012). The 17α-OHPreg levels significantly increased postoperatively in men (n = 9, *P* = .024).

**Conclusion:**

This study is the first detailed analysis of comprehensive steroid profiles in individuals with class II/III obesity and suggests that lower preoperative 17α-OHPreg levels are associated with insufficient weight loss after LSG.

The prevalence of obesity has been steadily increasing worldwide [1]. It is associated with an increased risk of various comorbidities, such as type 2 diabetes [2, 3]. Generally, obesity treatment involves behavioral interventions, nutrition, physical activity, pharmacotherapy, and metabolic (bariatric) procedures [4, 5]. Currently, metabolic surgery is one of the most effective treatments available [6], although inter-individual variations occur in weight loss outcomes [7, 8].

There are a few preoperative predictors of weight loss outcomes after metabolic surgery. Indeed, previous reports have suggested that a higher preoperative body mass index (BMI), older age, and the presence of type 2 diabetes, mental disorders, and a higher number of comorbidities are associated with insufficient weight loss [8-11], some of which are indications for metabolic surgery [12]. Therefore, it is important to identify the unknown factors associated with weight loss outcomes, which can help determine suitable candidates for undergoing metabolic surgery, in the context of emerging pharmacotherapies for obesity.

Accumulating evidence indicates that obesity influences steroid metabolism. For example, obesity induces cortisol dysregulation due to the activation of the hypothalamic-pituitary-adrenal axis [13] and 11β-hydroxysteroid dehydrogenase type 1 in adipose tissue [14, 15]. Obesity may also induce hypogonadism in men through the activation of aromatase in adipose tissue, where it converts testosterone to estrogen [16]. However, comprehensive blood steroid profiles, including those of intermediate metabolites, which are derived from the adrenal glands, gonads, and others [17, 18], have not been sufficiently studied. Few studies have reported blood steroid profiles determined using liquid chromatography–tandem mass spectrometry (LC-MS/MS) [19, 20], with limited steroid metabolites. Furthermore, the association between steroid profiles and weight loss outcomes after metabolic surgery remains unaddressed. Because steroid imbalances such as glucocorticoid excess and androgen deficiency play a role in the pathophysiology of obesity and its complications [21-23], inter-individual differences in obesity-related steroid profiles may affect the efficiency of weight loss. It is interesting to speculate that weight loss outcomes are associated with steroid metabolite(s), which can potentially predict weight loss outcomes in individuals with obesity.

Here we conducted plasma steroid profiling in individuals with class II/III obesity (BMI ≥ 35 kg/m^2^) using the newly developed LC-MS/MS method [24-27] to identify steroid metabolite(s) that may be related to weight loss outcomes after laparoscopic sleeve gastrectomy (LSG). This study is the first detailed analysis of comprehensive steroid profiles in individuals with class II/III obesity and suggests that lower preoperative 17α-hydroxypregnenolone (17α-OHPreg) levels are associated with insufficient weight loss after LSG.

## Materials and Methods

### Study Design and Subjects

This study was conducted as a retrospective observational study, based on clinical data extracted from existing medical records and stored blood samples obtained from individuals with class II/III obesity and healthy controls. The study consisted of 3 analyses: Analysis (i) was a cross-sectional comparison of steroid profiles between individuals with class II/III obesity and healthy controls; Analysis (ii) comprised analysis of steroid metabolites related to insufficient weight loss one year after LSG; and Analysis (iii) was a longitudinal investigation of changes in steroid profiles before and after LSG.

We included 120 individuals with class II/III obesity, who were admitted to Kyushu University Hospital between April 2018 and March 2023, and 15 healthy controls. A flowchart of participant selection is shown in Fig. S1 [28]. Exclusion criteria were the presence of malignancies (n = 15), undergoing steroid therapy (n = 8), history of hypopituitarism after pituitary surgery (n = 1), and absence of plasma samples for steroid profiling (n = 3).

For Analysis (i), we enrolled 93 individuals with class II/III obesity, who were followed up in our outpatient clinic until October 2024. Of them, 39 individuals underwent LSG, which is the most common type of metabolic surgery in Japan and is covered by the national health insurance. For Analysis (ii), 36 individuals with class II/III obesity and body weight records available 1 year after LSG were included (insufficient weight loss; n = 10 and sufficient weight loss; n = 26), after excluding 3 individuals with no records. For Analysis (iii), individuals from whom plasma samples were obtained after LSG were included (n = 20). Individuals without available postoperative plasma samples were excluded from the analysis. (n = 16). Plasma samples were collected at different times, with a median duration of 23.6 months (interquartile range [IQR], 10.2-33.1) after LSG.

This study was conducted in accordance with the Declaration of Helsinki and approved by the Institutional Ethics Committee (No. 2020-820) of our hospital. Informed consent was obtained from all participants.

### Diagnosis

Class II obesity was defined as 35 ≤ BMI < 40 kg/m^2^, and class III obesity as BMI ≥ 40.0 kg/m^2^, according to the World Health Organization criteria [29]. This level of obesity (BMI ≥ 35 kg/m^2^) corresponds to high-degree obesity as defined in Japan [5]. The healthy control group consisted of individuals with 18.5 ≤ BMI < 25.0 kg/m^2^ and no known medical conditions. Insufficient weight loss was defined as a percent total weight loss (%TWL) < 20%, while sufficient weight loss was defined as %TWL ≥ 20%. %TWL is widely used to evaluate weight loss outcomes because it is less influenced by preoperative weight [30]. Impaired glucose tolerance and type 2 diabetes were defined as 2-h plasma glucose level ≥ 140 mg/dL during the 75-g oral glucose tolerance test and a prior diagnosis as type 2 diabetes at baseline according to the criteria of the American Diabetes Association guidelines [31].

### Data Collection

Morning blood samples were obtained from all participants following an overnight fast, for performing general biochemical assays and steroid profiling. Plasma and serum samples were centrifuged and the supernatant was stored in a deep freezer (−80 °C) until analysis. Serum leptin concentration was measured using an enzyme-linked immunosorbent assay kit (Catalog # F10632101, RRID: AB_3697608; Cosmic Corporation Co., Ltd., Tokyo, Japan). In Analysis (ii), the baseline amount of body fat was measured in the fasted state using a bioelectrical impedance analysis device (InBody 770; InBody Japan Inc., Tokyo, Japan).

### Steroid Profiling

**Figure 1.**
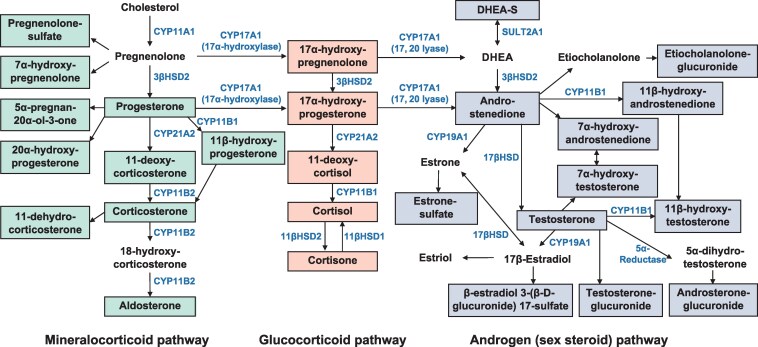
Steroid pathways of mineralocorticoids, glucocorticoids, and androgens (sex steroids). The steroid metabolites analyzed in this study are highlighted using rectangles. Abbreviations: DHEA, dehydroepiandrosterone; DHEA-S, dehydroepiandrosterone-sulfate.

Plasma steroid metabolites were measured using LC-MS/MS as previously described [24-27]. Initially, we measured 74 different steroid metabolites; however, after excluding those with high missing values, 27 were included in the study (Fig. 1).

All raw steroid profile data were normalized by log transformation, mean-centering, and scaling to unit variance. Missing values (1.7% of the total) were replaced with 1/5 of minimum positive values of their corresponding variables. Supervised partial least squares–discriminant analysis (PLS-DA) was performed to determine whether the 2 groups could be distinguished based on their steroid profiles. Variable importance in projection (VIP) scores derived from PLS-DA were calculated to rank the metabolites that contributed to group discrimination. VIP scores > 1 were considered statistically significant. Student's *t* test or paired *t* test was used to assess the statistical significance of each steroid metabolite between the 2 groups. Fold change (FC) analysis, expressed as log2(FC), was performed to compare the differences in mean concentrations of the respective steroid metabolites between individuals with class II/III obesity and healthy controls in Analysis (i).

### Statistical Analysis

To compare the characteristics of participants at baseline or after LSG, Fisher's exact test, Mann–Whitney *U*-test, and Wilcoxon signed-rank test were used, where appropriate. Multiple regression analysis was conducted to adjust for age in Analysis (i), and age, sex, glycated hemoglobin (HbA1c), presence of mental disorders, and preoperative BMI in Analysis (ii).

Test results were considered statistically significant at *P* values < .05. In case of multiple tests, a false discovery rate (FDR) < 0.1 was considered statistically significant. Statistical analyses, including plasma steroid profiling, were performed using MetaboAnalyst 6.0 [32] and R software (version 4.3.1).

## Results

### Analysis (i): Steroid Profiles of Individuals With Class II/III Obesity and Healthy Controls

The baseline characteristics are shown in Table 1. The median BMI of the groups was 40.91 (IQR: 37.12-47.03) kg/m^2^ for individuals with class II/III obesity and 21.09 (IQR: 19.5-21.85) kg/m^2^ for healthy controls (*P* < .001). The median age of the groups was 44 years (IQR: 40-52 years) for those with class II/III obesity and 33 years (IQR: 28.5-36 years) for healthy controls (*P* < .001). The median leptin concentrations of the groups were 41.6 (IQR: 26.8-61.8) ng/mL for those with class II/III obesity and 5.5 (IQR: 4.0-9.1) ng/mL for healthy controls (*P* < .001).

**Table 1.** Baseline characteristics of the participants

	Individuals with class II/III obesity			Healthy controls	*P* value*^a^*
	All	Men	Premenopausal women		
	n = 93	n = 45	n = 37	n = 15	
Age, years	44 (40-52)	46 (42-53)	41 (36-44)	33 (28.5-36)	<.001***
Male, no. (%)	48 (51.6)	45 (100)	0 (0)	8 (53.3)	.786
BMI, kg/m^2^	40.91 (37.12-47.03)	39.74 (36.64-52.03)	40.31 (37.12-44.57)	21.09 (19.5-21.85)	<.001***
Hypertension, no. (%)	72 (77.4)	34 (75.6)	27 (73)	0 (0)	<.001***
Impaired glucose tolerance or type 2 diabetes, no. (%)	74 (80.0)	35 (78)	30 (81)	0 (0)	<.001***
HbA1c, %	6.4 (5.9-7.7)	6.9 (5.9-7.7)	6.4 (5.8-7.7)		
FPG, mg/dL	102 (91-127)	104 (92-135)	100 (89-117)		
C-peptide, ng/mL	3.1 (2.4-3.9)	3.2 (2.6-4.7)	2.9 (2.4-3.5)		
TG, mg/dL	131 (95-214)	143 (96-219)	130 (94-188)		
LDL-C, mg/dL	114 (90-135)	104 (86-126)	119 (101-136)		
HDL-C, mg/dL	41 (35-48)	39 (32-43)	44 (39-52)		
SBP, mmHg	132 (124-143)	133 (121-143)	132 (126-145)		
DBP, mmHg	83 (76-92)	81 (75-92)	85 (80-92)		
Leptin, ng/mL	41.6 (26.8-61.8)	30.5 (15.7-45.7)	52 (36.9-65.0)	5.5 (4.0-9.1)	<.001***

Data are presented as median (interquartile range) or number (percentage).

Abbreviations: BMI, body mass index; DBP, diastolic blood pressure; FPG, fasting plasma glucose; HbA1c, glycated hemoglobin; HDL-C, high-density lipoprotein cholesterol; LDL-C, low-density lipoprotein cholesterol; SBP, systolic blood pressure; TG, triglycerides.

^a^Comparison between individuals with class II/III obesity and healthy controls.

^***^ *P* < .001.

**Figure 2.**
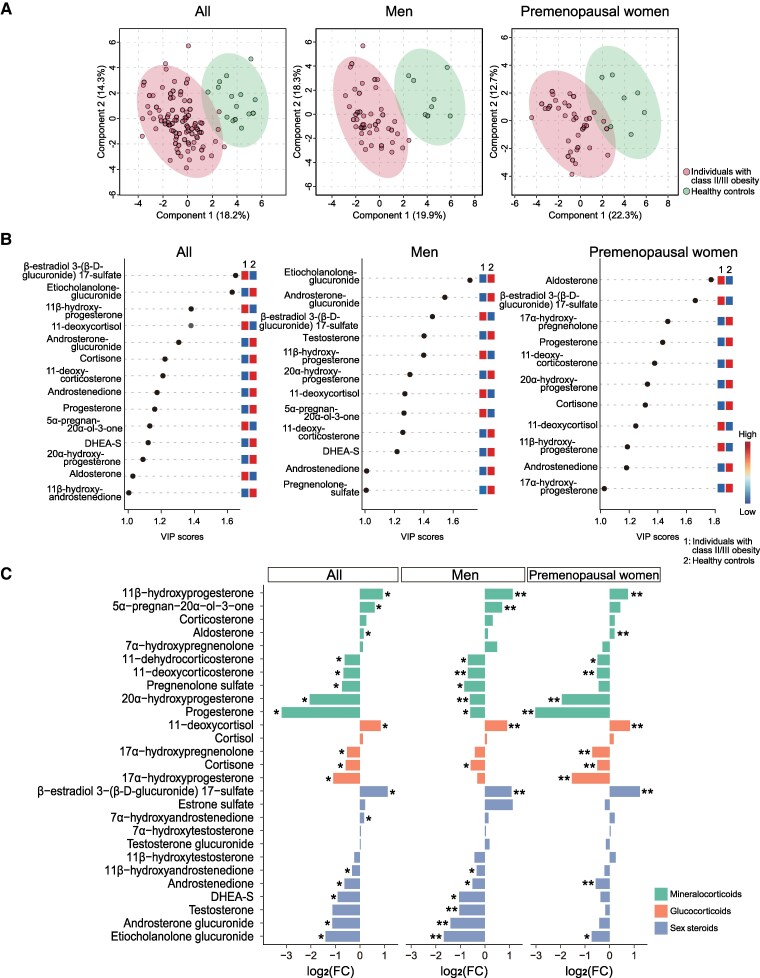
Differences in steroid profiles between individuals with class II/III obesity and healthy controls. (A) PLS-DA plots show distinct steroid profiles between individuals with class II/III obesity and healthy controls. Each point represents an individual sample. (B) Steroid metabolites ranked by VIP scores. Metabolites with VIP scores > 1 were considered significant factors contributing to the PLS-DA model. (C) Log2 values of fold change of each steroid metabolite, comparing individuals with class II/III obesity to healthy controls. *, FDR < 0.1. **, metabolites with VIP scores > 1 and FDR < 0.1 that showed significant differences after stratification by men and premenopausal women and adjusting for age. Abbreviations: DHEA-S, dehydroepiandrosterone-sulfate; FC, fold change; FDR, false discovery rate; PLS-DA, partial least squares–discriminant analysis; VIP, variable importance in projection.

PLS-DA was performed to clarify group differences in steroid profiles. The data showed a clear separation between individuals with class II/III obesity and healthy controls, suggesting that the 2 groups had different steroid profiles (Fig. 2A). Similar results were obtained when stratified by men and premenopausal women. Even when limited to individuals under 40 years of age, PLS-DA clearly distinguished the steroid profiles between those with class II/III obesity and healthy controls (Fig. S2) [28]. Figure 2B shows the ranking of steroid metabolites that contributed to the separation. In men, the top 4 metabolites included sex steroids, whereas in premenopausal women, aldosterone, β-estradiol 3-(β-D-glucuronide) 17-sulfate, and 17α-OHPreg were predominant. As shown in Fig. 2C, several metabolites were significantly different between those with class II/III obesity and healthy controls. After stratification by men and premenopausal women, metabolites with VIP > 1 (Fig. 2B) and FDR < 0.1 (Figure 2C) were considered significant (all metabolites with VIP > 1 had FDR < 0.1). Multiple regression analysis was performed on these metabolites to adjust for age, as there was a significant difference in the baseline age between those with class II/III obesity and healthy controls. The metabolites that remained significantly different between individuals with class II/III obesity and healthy controls, even after multiple regression analysis, are presented in Fig. 2C and listed in Tables S1 and S2 [28]. We found that 9 metabolites in men and 11 metabolites in premenopausal women were significantly different between individuals with class II/III obesity and healthy controls. In the mineralocorticoid pathway, premenopausal women with class II/III obesity exhibited significantly higher aldosterone and significantly lower progesterone levels than healthy controls, whereas for other metabolites, the trends were similar in men and premenopausal women. In the glucocorticoid pathway, cortisol levels did not differ between individuals with class II/III obesity and healthy controls; however, 11-deoxycortisol levels were significantly higher in both sexes among those with class II/III obesity, and the levels of other metabolites, including 17α-OHPreg, were significantly lower in premenopausal women than in healthy controls. In the sex steroid pathway, β-estradiol 3-(β-D-glucuronide) 17-sulfate levels were significantly higher in both sexes among those with class II/III obesity compared to healthy controls, whereas men had significantly lower androgen levels than healthy controls.

### Analysis (ii): Steroid Metabolites Related to Insufficient Weight Loss After LSG

Preoperative characteristics of individuals with class II/III obesity who experienced insufficient (%TWL < 20%) or sufficient (%TWL ≥ 20%) weight loss are shown in Table 2. The prevalence of mental disorders was significantly higher in the insufficient weight loss group (*P* = .010), whereas no significant differences were observed in other parameters, including leptin concentration.

**Table 2.** Baseline characteristics of the insufficient (%TWL < 20%) and sufficient (%TWL ≥ 20%) weight loss groups after LSG

	%TWL < 20%	%TWL ≥ 20%	*P* value
	n = 10	n = 26	
Age, years	47 (42.25-52.0)	44.0 (40.25-50.25)	.348
Male, no. (%)	4 (40)	14 (53.8)	.711
BMI, kg/m^2^	42.87 (39.62-47.68)	41.58 (37.33-48.89)	1.000
Body fat amount, kg	54.1 (46.3-59.4)	54.2 (42.6-79.2)	.737
Mental disorders, no. (%)	5 (50)	2 (7.7)	.010*
Hypertension, no. (%)	10 (100)	18 (69.2)	.076
Impaired glucose tolerance or type 2 diabetes, no. (%)	9 (90.0)	18 (69.2)	.392
HbA1c, %	7.2 (6.5-8.4)	6.0 (5.7-7.2)	.052
FPG, mg/dL	102 (98-129)	101 (91-117)	.560
C-peptide, ng/mL	3.0 (2.4-3.6)	3.7 (2.6-4.7)	.174
TG, mg/dL	115 (97-194)	119 (95-155)	.805
LDL-C, mg/dL	115 (91-132)	113 (95-126)	.986
HDL-C, mg/dL	46 (37-51)	40 (35-48)	.367
SBP, mmHg	135 (127-138)	132 (124-141)	.818
DBP, mmHg	85 (78-94)	83 (75-90)	.684
Leptin, ng/mL	32.5 (27.9-47.2)	49.9 (33.2-66.0)	.191
Leptin/body fat amount, ng/mL/kg	0.69 (0.46-0.80)	0.78 (0.68-1.03)	.244

Data are presented as median (interquartile range) or number (percentage).

Abbreviations: BMI, body mass index; DBP, diastolic blood pressure; FPG, fasting plasma glucose; HbA1c, glycated hemoglobin; HDL-C, high-density lipoprotein cholesterol; LDL-C, low-density lipoprotein cholesterol; LSG, laparoscopic sleeve gastrectomy; SBP, systolic blood pressure; TG, triglycerides; %TWL, percent total weight loss.

^*^ *P* < .05.

**Figure 3.**
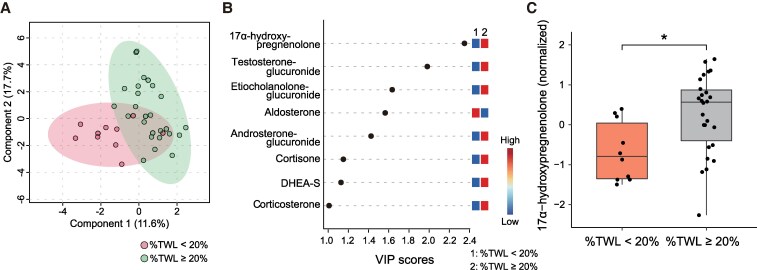
Differences in steroid profiles between the insufficient (%TWL < 20%) and sufficient (%TWL ≥ 20%) weight loss groups after LSG. (A) PLS-DA plots show the differences in steroid profiles between the 2 groups. Each point represents an individual sample. (B) Steroid metabolites ranked by VIP scores. Metabolites with VIP scores > 1 were considered significant factors contributing to the PLS-DA model. (C) Normalized levels of 17α-hydroxypregnenolone were compared between the 2 groups. *P < .05. Abbreviations: DHEA-S, dehydroepiandrosterone-sulfate; LSG, laparoscopic sleeve gastrectomy; PLS-DA, partial least squares–discriminant analysis; %TWL, percent total weight loss; VIP, variable importance in projection.

PLS-DA distinguished the insufficient and sufficient weight loss groups (%TWL < 20% and %TWL ≥ 20%, respectively), suggesting different preoperative steroid profiles between the 2 groups (Fig. 3A). The metabolite that contributed most significantly to the separation was 17α-OHPreg, with the highest VIP score of 2.35 (Fig. 3B). Preoperative 17α-OHPreg levels were significantly lower in the insufficient weight loss group than in the sufficient weight loss group (*P* = .011) (Fig. 3C). In multiple regression analysis, preoperative 17α-OHPreg levels were lower in the insufficient weight loss group, even after adjusting for age, sex, HbA1c, mental disorders, and preoperative BMI (*P* = .012) (Table 3).

**Table 3.** Multiple regression analysis: association between 17α-hydroxypregnenolone levels and variables

Variables	β	SE	*P* value
%TWL < 20%	−1.054	0.391	.012*
Age	0.024	0.016	.138
Male	0.055	0.292	.852
HbA1c	0.091	0.113	.427
Mental disorders	−0.243	0.424	.571
Preoperative BMI	−0.030	0.013	.034*

Abbreviations: BMI, body mass index; HbA1c, glycated hemoglobin; SE, standard error; %TWL, percent total weight loss.

^*^ *P* < .05.

### Analysis (iii): Longitudinal Investigation of Steroid Profiles in Individuals With Class II/III Obesity Before and After LSG

The characteristics before and after LSG in the longitudinal investigation are presented in Table S3 [28]. Median BMI decreased significantly from 40.43 (IQR: 36.86-45.73) to 29.25 (IQR: 27.92-33.06) kg/m^2^ (*P* < .001). Accordingly, the metabolic parameters significantly improved, and leptin concentrations were significantly reduced after LSG.

**Figure 4.**
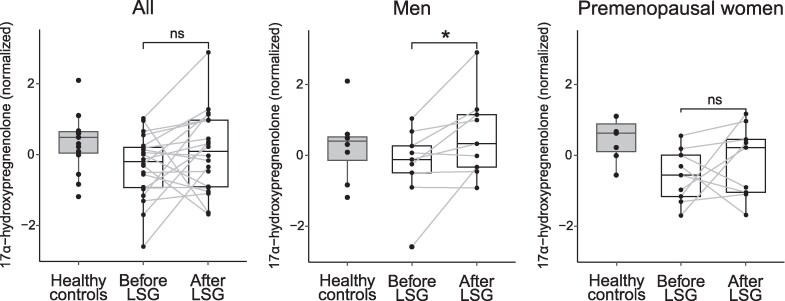
Changes in 17α-hydroxypregnenolone levels before and after LSG. Healthy controls are shown on the left for reference. *P < .05. The corresponding values in standard units (nmol/L) are as follows: Healthy controls, 540 (458-573); Before LSG, 419 (320-486); After LSG, 506 (322-646); Men: Healthy controls, 523 (433-547); Before LSG, 430 (374-495); After LSG, 506 (397-686); Premenopausal women: Healthy controls, 568 (470-628); Before LSG, 367 (294-452); After LSG, 490 (307-532). Data are presented as median (interquartile range). Abbreviations: LSG, laparoscopic sleeve gastrectomy.

PLS-DA was used to distinguish steroid profiles in individuals with class II/III obesity before and after LSG (Fig. S3) [28]. Following LSG, the 17α-OHPreg level in men significantly increased to levels similar to that observed in healthy controls (*P* = .024). Similarly, it tended to increase postoperatively in premenopausal women (Fig. 4).

## Discussion

In this study, we examined the steroid profiles of individuals with class II/III obesity, steroid metabolite(s) associated with insufficient weight loss after LSG, and their changes before and after LSG, and obtained the following findings. (i) Individuals with class II/III obesity exhibited steroid profiles different from those of healthy controls, and significant differences were found in 9 and 11 metabolites in men and premenopausal women, respectively. (ii) Distinct preoperative steroid profiles were observed for the insufficient and sufficient weight loss groups after LSG; preoperative 17α-OHPreg was the most significant factor contributing to the distinction. Notably, preoperative 17α-OHPreg levels were significantly lower in the insufficient weight loss group than in the sufficient weight loss group, even after adjusting for confounders. (iii) Among individuals with class II/III obesity, lower preoperative 17α-OHPreg levels showed a tendency to improve after LSG.

Our data suggest that compared to healthy men, those with obesity have lower testosterone levels, which is consistent with the findings of previous studies [33-35]. Our study found lower progesterone levels in premenopausal women with class II/III obesity compared to healthy controls, which aligns with previous findings and may result from impaired LH pulse amplitude with a shorter luteal phase in women with obesity [36, 37]. In agreement with previous reports, we found higher aldosterone and lower 17α-hydroxyprogesterone levels in premenopausal women with class II/III obesity compared to healthy controls [20, 38-40]. No significant difference in circulating cortisol levels between individuals with class II/III obesity and healthy controls is consistent with previous reports indicating that cortisol dysregulation primarily occurs at the tissue level and is not reflected in circulating morning cortisol concentrations [14, 15]. As described above, the steroid metabolite data obtained in this study are roughly comparable to those reported previously, thereby validating the steroidomics analysis in this study.

This study provides evidence for previously unrecognized difference in steroid metabolites between individuals with class II/III obesity and healthy controls, suggesting that the associated steroidogenic enzyme activities are altered in individuals with obesity. Circulating androgens are primarily derived from the testes and adrenal glands of men, and the adrenal glands and ovaries of women, whereas circulating mineralocorticoids and glucocorticoids are mainly produced in the adrenal cortex [17, 18]. Alterations in steroidogenic enzyme activity in each organ in individuals with obesity remain unclear, highlighting the importance of this study. Given the substantial differences in fat mass among individuals with class II/III obesity, healthy controls, and those who underwent LSG, alterations in the activities of steroidogenic enzymes in adipose tissue, such as aromatase, may have further contributed to the observed differences.

We found that preoperative 17α-OHPreg is a key steroid metabolite associated with insufficient weight loss after LSG. The 17α-OHPreg levels tended to increase postoperatively, similar to the levels in healthy controls. These observations, together with weight loss after LSG, support the concept that 17α-OHPreg is a critical metabolite associated with obesity. 17α-OHPreg is an intermediate metabolite produced from pregnenolone by CYP17A1 and is a precursor of cortisol and sex steroids. However, the reasons for its association with obesity and weight loss, and the specific tissues involved, remain unclear.

As a possible underlying mechanism, individual differences in *CYP17A1* gene expression may contribute to the observed differences in 17α-OHPreg levels. Previous studies have demonstrated that *CYP17A1* knockout mice develop obesity and metabolic disorders [41, 42]. These findings suggest that CYP17A1 may play a role in the pathophysiology of obesity, and that decreased expression of *CYP17A1*—potentially leading to reduced levels of 17α-OHPreg—may be associated with obesity and postoperative weight loss outcomes.

Moreover, some studies suggest that elevated leptin levels may alter activities of steroidogenic enzymes including CYP17A1 [43, 44], thereby leading to reduced 17α-OHPreg levels. Individuals with class II/III obesity exhibited higher leptin levels and lower 17α-OHPreg levels than healthy controls. After LSG, leptin levels decreased and 17α-OHPreg levels tended to increase. These findings may reflect leptin-induced alterations in steroidogenic enzyme activity. However, while 17α-OHPreg levels were lower in the insufficient weight loss group than the sufficient weight loss group, there was no significant difference in leptin levels between the 2 groups. Although this may be partly attributable to the small sample size, other contributing factors may also have influenced the results.

17α-OHPreg may function not only as a steroidogenic precursor but also as a biologically active ligand. In this study, no significant associations were observed between insufficient weight loss and the levels of cortisol or sex hormones, which are active downstream steroids of 17α-OHPreg and are known to be strongly associated with obesity. A recent study reported that 17α-OHPreg inhibits metabolic dysfunction–associated steatohepatitis via the G protein–coupled receptor GPR56 [45]. While GPR56 has been implicated in the regulation of adipogenesis [46], the role of 17α-OHPreg and the impact of GPR56 on obesity remain unclear, and further studies are warranted.

This study had several limitations. First, as this was a retrospective observational study, causality could not be established. Second, this study was conducted at a single center with a relatively small and imbalanced sample size. The subgroup analyses of men and premenopausal women in Analysis (i), as well as the comparison in Analysis (ii), demonstrated statistical power below the conventional threshold of .8. As this may have increased the risk of Type II error, the nonsignificant findings from these analyses should be interpreted with caution. Future studies with larger sample sizes and balanced group distributions will be necessary to validate the findings from these analyses. Third, the study included participants with class II/III obesity, many of whom had been treated with angiotensin-converting enzyme inhibitors, angiotensin II receptor blockers, and/or mineralocorticoid receptor blockers, which may have affected steroid metabolism. Fourth, this study could not account for the different menstrual cycle phases as it was difficult to define follicular or luteal phases because of the high prevalence of menstrual irregularities in individuals with class II/III obesity [47].

In conclusion, this study is the first detailed analysis of comprehensive steroid profiles in individuals with class II/III obesity and suggests that preoperative 17α-OHPreg levels are associated with weight loss outcomes after LSG. Steroid metabolism in obesity remains largely unexplored and our findings offer novel insights into this field. Our data suggest that 17α-OHPreg may serve as a potential biomarker for predicting weight loss outcomes after metabolic surgery. The development and widespread availability of a simple method for measuring this metabolite could offer practical clinical utility for supporting surgical decision-making and postoperative follow-up, when combined with other known predictive factors.

## Data Availability

Some or all datasets generated and/or analyzed during the current study are not publicly available but are available from the corresponding author on reasonable request.

## Current Affiliation

Yayoi Matsuda current affiliation is Department of Diabetology, Fukuoka Saiseikai Futsukaichi Hospital. Eiji Oki current affiliation is Department of Advanced Medicine and Innovative Technology, Kyushu Universit Hospital.

## Abbreviations

17α-OHPreg: 17α-hydroxypregnenolone
BMI: body mass index
FDR: false discovery rate
HbA1c: glycated hemoglobin
IQR: interquartile range
LC-MS/MS: liquid chromatography–tandem mass spectrometry
LSG: laparoscopic sleeve gastrectomy
PLS-DA: partial least squares–discriminant analysis
%TWL: percent total weight loss
VIP: variable importance in projection
